# Nanoparticles – known and unknown health risks

**DOI:** 10.1186/1477-3155-2-12

**Published:** 2004-12-08

**Authors:** Peter HM Hoet, Irene Brüske-Hohlfeld, Oleg V Salata

**Affiliations:** 1 Katholieke Universiteit Leuven, Pneumologie, Longtoxicologie, Campus GHB, Herestraat 49, Leuven B-3000, Belgium; 2 GSF-Forschungszentrum für Umwelt und Gesundheit, GmbH Ingolstädter Landstraß1, D-85764 Neuherberg, Germany; 3 Sir William Dunn School of Pathology, University of Oxford, South Parks Road, Oxford OX1 3RE, UK

## Abstract

Manmade nanoparticles range from the well-established multi-ton production of carbon black and fumed silica for applications in plastic fillers and car tyres to microgram quantities of fluorescent quantum dots used as markers in biological imaging. As nano-sciences are experiencing massive investment worldwide, there will be a further rise in consumer products relying on nanotechnology. While benefits of nanotechnology are widely publicised, the discussion of the potential effects of their widespread use in the consumer and industrial products are just beginning to emerge. This review provides comprehensive analysis of data available on health effects of nanomaterials.

## 1. Introduction

Scientists world-wide are continuing to discover unique properties of everyday materials at the sub micrometer scale [1,2]. This size domain is better known as nano- (a billionth) meter domain. These novel properties of common materials observable only at the nano-scale dimensions have already found their first commercial applications [3]. For example, nanomaterials are present in some sunscreens, toothpastes, sanitary ware coatings and even food products. Manmade nanoparticles ranges from the well-established multi-ton production of carbon black and fumed silica for applications in plastic fillers and car tyres to microgram quantities of fluorescent quantum dots used as markers in biological imaging. As nano-sciences are experiencing massive investment worldwide [4,5], there will be a further rise in consumer products relying on nanotechnology [6].

While benefits of nanotechnology are widely publicised, the discussion of the potential effects of their widespread use in the consumer and industrial products are just beginning to emerge [7,8]. Both pioneers of nanotechnology [9] and its opponents [10] are finding it extremely hard to argue their case as there is limited information available to support one side or the other. It has been shown that nanomaterials can enter the human body through several ports. Accidental or involuntary contact during production or use is most likely to happen via the lungs from where a rapid translocation through the blood stream is possible to other vital organs [11]. On the cellular level an ability to act as a gene vector has been demonstrated for nanoparticles [12]. Carbon black nanoparticles have been implicated in interfering with cell signalling [13]. There is work that demonstrates uses of DNA for the size separation of carbon nanotubes [14]. The DNA strand just wraps around it if the tube diameter is right. While excellent for the separation purposes it raises some concerns over the consequences of carbon nanotubes entering the human body.

In this review we summarise the known facts about nanomaterial hazards, discuss the potential entry points of nanoparticles into the human body, explore their likely pathways inside the body and analyse published experimental results on the bioactivity of nanomaterials.

## 2. General background

Human skin, intestinal tract and lungs are always in direct contact with the environment. Whereas skin acts as a barrier, lungs and intestinal tract also allow transport (passive and/or active) of various substances like water, nutrients or oxygen. Because of that fact they are likely to be a first port of entry for nanomaterials journey into the human body. Our knowledge in this field mainly comes from drug delivery (pharmaceutical research) and toxicology (xenobiotics) studies. Human skin functions as a strict barrier and no essential elements are taken up through the skin (except radiation necessary to build up vitamin D). The lungs exchange oxygen and carbon dioxide with the environment, and some water escapes with warm exhaled air. The intestinal tract is in close contact with all the materials taken up orally; there all nutrients (except gasses) are exchanged between the body and the environment.

**Figure 1:**
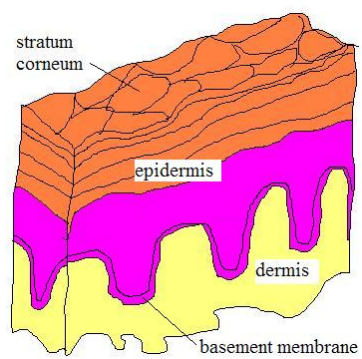
schematic representation of human skin; Stratum corneum is the top of the five layers making epidermis, it is composed of keratinised dead cells glued by lipids. It is shed off and replaced every two weeks. Depending on the part of the body its thickness varies from 0.05 mm to 1.5 mm.

The histology of the environmental contact sides of these three organs is significantly different. The skin of an adult human is roughly 1.5 m^2 ^in area, and is at most places covered with a relatively thick first barrier (10 micron) which is build of strongly keratinised dead cells (Fig 1). This first barrier is difficult to pass for ionic compounds as well as water soluble molecules.

**Figure 2:**
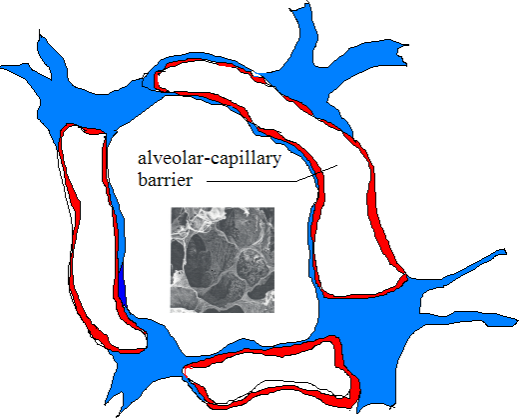
Cross-section of alveoli; Schematic cross-section of alveoli showing a very thin (500 nm) separation between blood and air. An SEM image of the alveoli is shown in the inset.

The lung consists of two different parts, airways (transporting the air in and out the lungs) and alveoli (gas exchange areas). Human lungs contain about 2300 km of airways and 300 million alveoli (gas exchange areas) (Fig 2). The surface area of the lungs is 140 m^2 ^in adults, as big as a tennis court. The airways are a relatively robust barrier, an active epithelium protected with a viscous layer of mucus. In the gas exchange area, the barrier between the alveolar wall and the capillaries is very thin. The air in the lumen of the alveoli is just 0.5 micron away from the blood flow. The large surface area of the alveoli and the intense air-blood contact in this region makes the alveoli less well protected against environmental damage when compared with airways.

**Figure 3:**
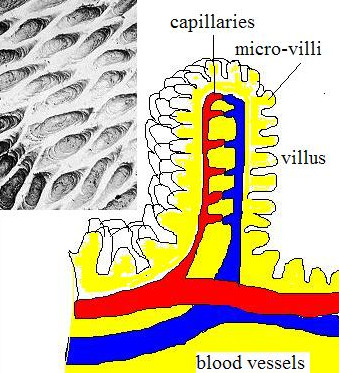
Villi in small intestine; A surface structure of villi covered with micro-villi is dramatically multiplies the area of gastero-intestine tract to 200 m^2^. Inset shows an SEM image of villi.

The intestinal tract is a more complex barrier – exchange side, it is the most important portal for macromolecules to enter the body. From the stomach, only small molecules can diffuse through the epithelium. The epithelium of the small and large intestines is in close contact with ingested material so that nutrients can be utilized. A mixture of disaccharides, peptides, fatty acids, and monoglycerides generated by digestion in small intestine are further transformed and taken in the villi (Fig 3). Villi, in turn, are covered with micro-villi, which bring overall surface available to nutrients to 200 square meters.

## 3. Lung

### 3.1 Inhalation and pulmonary clearing of insoluble solids

The pathogenic effects of inhaled solid material depend primarily on achieving a sufficient lung burden [15]. The lung burden is determined by the rates of deposition and clearance. Logically, for any dust or fibre, a steady-state dose level will be achieved when the rates come into balance. This is only true when the solid material does not interfere with the clearance mechanisms. In respect to the burden the chemical and physical properties of the material itself are important insofar as they influence deposition and clearance rates. Spherical solid material can be inhaled when its aerodynamic diameter is less than 10 micron. The smaller the particulates the deeper they can travel into the lung, particles smaller than 2.5 micron will even reach the alveoli. Ultrafine particles (nanoparticles with an aerodynamic diameter of less than 100 nm) are deposited mainly in the alveolar region. Fibres are defined as solid materials with a length to diameter ratio of at least 3:1. Their penetration into the lung depends on the aerodynamic properties. Fibres with a small diameter will penetrate deeper into the lungs, while very long fibres (>>20 micron) are predominantly stuck in the higher airways [16-21].

The mucociliary escalator dominates the clearance from the upper airways; clearance from the deep lung (alveoli) is predominantly by macrophage phagocytosis. The mucociliary escalator is an efficient transport system pushing the mucus, which covers the airways, together with the trapped solid materials towards the mouth. The phagocytosis of particles and fibres results in activation of macrophages and induces the release of chemokines, cytokines, reactive oxygen species, and other mediators; this can result in sustained inflammation and eventually fibrotic changes. The phagocytosis efficiency can be affected by the (physical-chemical) characteristics of the solid material (see below); moreover, fibres too long to be phagocytized (fibres longer than the diameter of the alveolar macrophage) will only be cleared very slowly.

Laboratory exposure studies have shown that if the inhaled concentrations are low, such that the deposition rate of the inhaled particles is less than the mechanical alveolar macrophage-mediated clearance rate in the lung, then the retention half time is about 70 days (steady-state lung burden during continuous exposure). If the deposition rate of the inhaled particles exceeds this clearance rate, the retention half time is significantly increased, reflecting an impaired or prolonged alveolar macrophage-mediated clearance function with continued accumulation of lung burden (overload). Inhaled fibres, which are persistent in the alveoli, can interact with the pulmonary epithelial cells or even penetrate the alveolar wall and enter the lung tissue. These fibres are often described as being in the "interstitial" where they may lie between or within the cells making up the alveolar walls. Bio-persistent solid materials, certainly those particles containing mutagenic substances or asbestos fibres or silica, which remain for years in the lungs, increase the risk of developing cancer.

### 3.2 Deposition and clearing of solid nanomaterials

It has been reported recently that nanotubes show a sign of toxicity [22], confirmed in two independent publications by Warheit *et al *[23] and Lam *et al *[24], which demonstrated the pulmonary effects of single walled cabon nanotubes in vivo after intratracheal instillation, in both rats and mice. Both groups reported granuloma formation, and some interstitial inflammation. The research group of Warheit et al [23] concluded that these findings (multifocal granulomas) may not have physiological relevance, and may be related to the instillation of a bolus of agglomerated nanotubes. But for the authors of [24] their results indicate that if carbon nanotubes reach the lungs, they are much more toxic than carbon black and can be more toxic than quartz. These studies have to be read with some caution because a study by the National Institute for Occupational Safety and Health (NIOSH) showed that none or only a small fraction of the nanotubes present in the air can be inhaled [25].

Clearance from the lung depends not only on the total mass of particles inhaled but also on the particle size and, by implication, on particle surface, as shown in the following studies. A sub-chronic 3 months inhalation exposure of rats to ultrafine (~20 nm) and fine (~200 nm) titanium dioxide (TiO2) particles demonstrated that the ultrafine particles cleared significantly slower, showed more translocation to interstitial sites and to regional lymph nodes when compared to the fine TiO2 particles [26]. By comparing carbon black particles of similar size and composition but with significant specific surface area difference (300 versus 37 m^2^/g), it was found that the biological effects (inflammation, genotoxicity, and histology) were dependent on specific surface area and not particle mass. Similar findings were reported in earlier studies on tumorigenic effects of inhaled particles. It was shown that tumour incidence correlated better with specific surface area than with particle mass [27,28].

Comparing the health effects of chronically inhaled TiO2 particles with distinctly different sizes, it is remarkable that the low exposure (10 mg/m^3^) study [29] resulted in a greater lung tumour incidence than the high exposure (250 mg/m^3^) study [30]. The inhaled particles in both studies consisted of aggregated primary particles, with an aerodynamic diameter that was probably not very different. The primary particle size of the low dose study was 20 nm, while it was approximately 300 nm in the latter study.

In summary, most nano-sized spherical solid materials will easily enter the lungs and reach the alveoli. These particles can be cleared from the lungs, as long as the clearance mechanisms are not affected by the particles themselves or any other cause. Nano-sized particles are more likely to hamper the clearance resulting in a higher burden, possibly amplifying any possible chronic effects caused by these particles. It is also important to note that specific particle surface area is probably a better indication for maximum tolerated exposure level than total mass. Inhaled nano-fibres (diameter smaller than 100 nm) also can enter the alveoli and their clearing would, in addition, depend on the length of the specific fibre. Recent publications on the pulmonary effects of carbon nanotubes confirm the intuitive fear that nano-sized fibre can induce a rather general non-specific pulmonary response.

### 3.3 Particle surface and biocompatibility

Reports on the surface properties of nanoparticles, both physical and chemical, stress that nanoparticles differ from bulk materials. Their properties depend heavily on the particle size. Therefore, nanoparticles are not merely small crystals but an intermediate state of matter placed between bulk and molecular material. Independently of the particle size, two parameters play dominant role. The charges carried by the particle in contact with the cell membranes and the chemical reactivity of the particle [31].

#### 3.3.1 Surface charges

Polycationic macromolecules show a strong interaction with cell membranes in vitro. A good example can be found in the Acramin F textile paint system. Three poly-cationic paint components exhibited considerable cytotoxicity (LD50 generally below 100 mg/ml for an incubation of 20–24 hours) in diverse cell cultures, such as primary cultures of rat and human type II pneumocytes, and alveolar macrophages and human erythrocytes. The authors argued that the multiple positive charges play an important role in the toxic mechanism [32,33]. Biocompatibility studies [34] revealed that the cytotoxicity of polycationic materials such as DEAE-dextran and poly-L-lysine (PLL) [35,36], dendrimers [37] and polyethylenimine (PEI) [38] increases with the increase in their molecular weight. However, these findings apply only to polymers having same chemical structure, but not for different types of polycations. Consequently, to explain the toxicity of polymers with different structures further parameters have to be taken into account.

Dekie *et al *[39] concluded that the presence of a primary amine group on poly L-glutamic acid derivatives has a significant toxic effect on red blood cells causing them to agglutinate. Not only the type of amino function but also the charge density resulting from the number and special arrangement of the cationic residues is an important factor for cytotoxicity. Ryser [40] suggested that a three-point attachment is necessary for eliciting a biological response on cell membranes, and argued that the activity of a polymer will decrease when the space between reactive amine groups is increased. The arrangement of cationic charges depends on the three-dimensional structure and flexibility of the macromolecules and determines the accessibility of their charges to the cell surface. For example, branched molecules were found to be more efficient in neutralising the cell surface charge than polymers with linear or globular structure, as rigid molecules have more difficulties to attach to the membranes than flexible molecules [41]. Therefore, high cationic charge densities and highly flexible polymers should cause higher cytotoxic effects than those with low cationic charge densities. Globular polycationic macromolecules (cationised Human Serum Albumine (cHSA), ethylenediamine-core poly(amidoamine) dendrimers (PAMAM) were found to be polymers with a good biocompatibility (low cytotoxicity), whereas polymers with a more linear or branched and flexible structure (e.g. polydiallyldimethylammonium chloride (DADMAC), PLL, PEI) showed higher cell damaging effects.

#### 3.3.2 The surfactant interaction and surface chemistry

Geiser *et al *[42] studied the influence of the particle surface chemistry on its interaction with the lung's surface-lining layer. They found that, regardless of the nature of their surfaces, particles will be submersed into the lining layer after their deposition in small airways and alveoli. This displacement is promoted by the surfactant film itself, whose surface tension falls temporarily to relatively low values [42,43]. On the other hand, reactive groups on a particle surface will certainly modify the biological effects. For silica, it has been shown that surface modification of quartz affects its cytotoxicity, inflammogenicity and fibrogenicity. These differences are mainly due to particle surface characteristics [44]. Specific cytotoxicity of silica is strongly correlated with the appearance of surface radicals and reactive oxygen species (ROS), which is considered to be the key event in the development of fibrosis and lung cancer by this compound [45].

Although the type of particle does not seem to play an important role in whether it is embedded in the surfactant lining of the alveoli, the embedding process itself is crucial. Particle-cell interaction is possible only after the immersion of the particulates in the lining fluid and research is needed to study this phenomenon in detail in relation to inhaled nanoparticles. Logically, as described in the report for silica [45], the reactive groups on nanoparticles influence their interaction with the lung (or more general with biological material). In some instances it might be possible to predict the reactivity of the nano-surface. However, considering the scarcity of data, it would be sensible to verify these predictions by some laboratory testing.

### 3.4 Systemic translocation of inhaled particles

The impact of inhaled particles on other organs has only recently been recognised. Most research has concentrated on the possible consequences of particle related malfunction of the cardio-vascular system, such as arrhythmia, coagulation [46] etc. However, recent data support the concept that the autonomic nervous system may also be a target for the adverse effects of inhaled particulates [47,48,11]. Two complementary hypotheses explain the cardiovascular malfunctions after inhalation of ultra-fine particles. The first hypothesis explains the observed effects by the occurrence of strong (and persistent) pulmonary inflammatory reactions in the lungs, leading to the release of mediators (see above), which may influence the heart, coagulation, or other cardiovascular endpoints. The second hypothesis is that the particles translocate from the lungs into the systemic circulation and thus, directly or indirectly, influence haemostasis or cardiovascular integrity.

**Figure 4:**
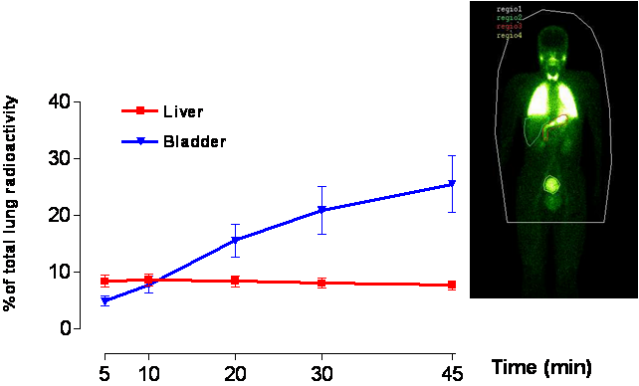
Translocation of inhaled ultrafine particles. Time-activity curve over liver and bladder expressed as percent of initial lung radioactivity. Insert, Whole body gamma camera image of 1 representative volunteer recorded at 60 minutes. The radioactivity over the organs is expressed as counts per minute (CPM) per pixel within each region of interest (ROI). The values recorded over the stomach were not included because this radioactivity may also come partly from swallowing of particles deposited in the mouth. Reproduced with permission from Nemmar *et al*, "Passage of inhaled particles into the blood circulation in humans", Circulation 2002;105(4):411-41.

In the evaluation of the health effects of inhaled nanoparticles the translocation to the systemic circulation is an important issue. Conhaim and co-workers [49] found that the lung epithelial barrier was best fitted by a three-pore-sized model, including a small number (2%) of large-sized pores (400-nm pore radius), an intermediate number (30%) of medium-sized pores (40-nm pore radius), and a very large number (68%) of small-sized pores (1.3-nm pore radius). The exact anatomical location of this structure, however, remains to be established (see the review by Hermans and Bernard [50]). Until recently, the possible passage of xenobiotic particles has not been attracting much attention, although, the concept is now gaining acceptance in pharmacology for the administration of macromolecular drugs by inhalation [51]. Nemmar *et al *[11] studied the particle-translocation of inhaled ultrafine technetium (^99m^Tc) labelled carbon particles to the blood. These particles, which are very similar to the ultrafine fraction of actual pollutant particles, diffused rapidly – within 5 minutes – into the systemic circulation (Fig 4). The authors concluded that phagocytosis by macrophages and/or endocytosis by epithelial and endothelial cells are responsible for particle-translocation to the blood but other roots must also exist.

The literature on the translocation of very small particles from the lungs into the blood circulation is limited and often conflicting. A recent study has reported deposition and clearance over 2 h of an ultrafine (60 nm) ^99m^Tc labelled aerosol in human volunteers. No significant radioactivity was found in the liver (1–2 % of the inhaled radioactivity) but, unfortunately, no radioactivity measurements with blood were reported [52]. In agreement with findings of Nemmar et al [11], Kawakami et al. [53] have reported the presence of radioactivity in blood immediately after inhalation of 99mTc-technegas in human volunteers. It is also known [54] that aerosolised insulin gives a rapid therapeutic effect although the pathways for this translocation are still unclear. In addition to human studies, in experimental animal studies, we [11] and others [55,16,57] have reported extra-pulmonary translocation of ultrafine particles after intra-tracheal instillation or inhalation. However, the amount of ultrafine particles that translocate into blood and extra-pulmonary organs differed among these studies. It has also been shown that, following intranasal delivery, polystyrene microparticles (1.1 micron) can translocate to tissues in the systemic compartment [58]. A recent study [59] has provided, for the first time, morphological data showing that inhaled polystyrene particles are transported into the pulmonary capillary space, presumably by trans-cytosis. Another alley of translocation from the lungs towards other organs has been undertaken by Oberdörster *et al *[19]. In inhalation experiments with rats, using 13C-labelled particles, they found that nano-sized particles (25 nm) were present in several organs 24 hours after exposure. The most extraordinary finding was the discovery of particles in the central nervous system (CNS). The authors examined this phenomenon further and found that particles, after being taken up by the nerve cells, can be transported via nerves (in this experiment via the olfactory nerves) at a speed of 2.5 mm per hour [56].

Passage of solid material from the pulmonary epithelium to the circulation seems to be restricted to nanoparticles. The issue of particle translocation still need to be clarified: both the trans-epithelial transport in the alveoli and the transport via nerve cells. Thus, the role of factors governing particle translocation such as the way of exposure, dose, size, surface chemistry and time course should be investigated. For instance, it would be also very important to know how and to what extent lung inflammation modulates the extra-pulmonary translocation of particles.

### 3.5 Fibre bio-persistence

Long non-phagocytizable fibres (in humans longer than 20 micron) will not be effectively cleared from the respiratory tract. The main determinants of fibre bio-persistence are species specific physiological clearance and fibre specific bio-durability (physical-chemical processes). In the alveoli the rate at which fibres are physically cleared depends on the ability of alveolar macrophages to phagocytose them. Macrophages containing fibres longer than their own diameter may not be mobile and be unable to clear the fibres from the lung. The bio-durability of a fibre depends on dissolution and leaching as well as mechanical breaking and splitting. Long fibres in the lung can disintegrate, leading to shorter fibres that can be removed by the macrophages. Bio-persistent types of asbestos, where breakage occurs longitudinally, result in more fibres of the same length but smaller diameter. Amorphous fibres break perpendicular to their long axis [60,61], resulting in fibres that can be engulfed by the macrophages.

It is self-evident that the slower the fibres are cleared (high bio-persistence), the higher is the tissue burden and the longer the fibres reside in a tissue the higher is the probability of an adverse response. A milestone was set by Stanton et al [62,63] who undertook a series of experiments with 17 samples of carefully sized fibrous glass. They found that for mesothelioma induction in rats, the peak activity was in the fibres greater than 8 micron in length and less than 1.3 micron in diameter. These findings are known as the "Stanton hypothesis". However these results do not strictly indicate that all fibres longer than the lower threshold are equally active or that shorter fibres are not, although fibres less than 5 micron in length did not appear to contribute to lung cancer risk in exposed rats [64]. Risk appears to increase with length, with fibres more than 40 micron in length imposing the highest risk. For the recent review see Schins [65].

The bio-durability of fibres with a diameter < 100 nm will probably not differ from larger inhalable fibres. Therefore, great caution must be taken in case of the contact with nano-fibres, Bio-durability tests must be performed before releasing any products containing them. Carbon nanotubes, which are of high technical interest, are one of the materials which need to be tested in depth concerning bio-persistence and cancer risk. The first toxicological studies indicated that carbon nanotubes can be a risk for human health [22-24], while exposure assessment did indicate that these materials are probably not inhaled [25].

## 4. Intestinal tract

Already in 1926 it was recognised by Kumagai [66] that particles could translocate from the lumen of the intestinal tract via aggregations of intestinal lymphatic tissue (Peyer's patches (PP)), containing M-cells (specialised phagocytic enterocytes). Particulate uptake happens not only via the M-cells in the PP and the isolated follicles of the gut-associated lymphoid tissue, but also via the normal intestinal enterocytes. There have been a number of excellent reviews on the subject of intestinal uptake of particles [51,66]. Uptake of inert particles has been shown to occur trans-cellularly through normal enterocytes and PP via M-cells, and to a lesser extent across para-cellular pathways [67]. Initially it was assumed that the PP did not discriminate strongly in the type and size of the absorb particles. Later it has been shown that modified characteristics, such as particle size [68] the surface charge of particles [69], attachment of ligands [70,71] or coating with surfactants [72], offers possibilities of site-specific targeting to different regions of the gastro intestine tract (GIT), including the PP [73].

The kinetics of particle translocation in the intestine depends on diffusion and accessibility through mucus, initial contact with enterocyte or M-cell, cellular trafficking, and post-translocation events. Charged particles, such as carboxylated polystyrene nanoparticles [69] or those composed of positively charged polymers exhibit poor oral bioavailability through electrostatic repulsion and mucus entrapment. Szentkuti [74] determined the rate of particle diffusion across the mucus layer to the enterocyte surface with respect to both size and surface charge of the particles. In brief, Szentkuti [74] observed that cationic nanometer-sized latex particles became entrapped in the negatively charged mucus, whereas repulsive carboxylated fluorescent latex nanoparticles were able to diffuse across this layer. The smaller the particle diameter the faster they could permutate the mucus to reach the colonic enterocytes; 14 nm diameter permeated within 2 min, 415 nm particles took 30 min, while 1000-nm particles were unable to translocate this barrier. Within, the time of the experiment (30 min) none of the particles was endocytosed by the enterocytes despite the fact that the latex nanoparticles preferentially bound the cell surface more strongly than the mucus. After a longer time window (oral gavage for several days) a sparse accumulation of charged particulates in the lamina propria (connective tissue under the epithelia) was found compared to uncharged latex nanoparticles in the same size range [69].

Particulates, once in the sub-mucosal tissue, are able to enter both lymphatic and capillaries. Particles entering the lymphatic are probably important in the induction of secretory immune responses while those which enter the capillaries become systemic and can reach different organs. In one study [75], the body distribution after translocation of polystyrene particles was examined in some detail. Polystyrene spheres (ranging from 50 nm to 3 micron) were fed by gavage to female Sprague Dawley rats daily for 10 days at a dose of 1.25 mg/kg. As much as 34 % and 26% of the 50 and 100 nm particles were absorbed respectively. Those larger than 300 nm were absent from blood. No particles were detected in heart or lung tissue.

### 4.1 Intestinal Translocation and Disease

Crohn's disease is characterised by transmural inflammation of the gastrointestinal tract. It is of unknown aetiology, but it is suggested that a combination of genetic predisposition and environmental factors play a role. Particles (0.1–1.0 micron) are associated with the disease and indicated as potent adjuvants in model antigen-mediated immune responses. In a double-blind randomised study, it has been shown that a particle low diet (low in calcium and exogenous microparticles) alleviates the symptoms of Crohn's disease [76]. Although there is a clear association between particle exposure and uptake and Crohn's disease, little is known of the exact role of the phagocytosing cells in the intestinal epithelium. It has been suggested that the disruption of the epithelial barrier function by apoptosis of enterocytes is a possible trigger mechanism for mucosal inflammation. The patho-physiological role of M cells is unclear; e.g., it has been found that in Crohn's disease M cells are lost from the epithelium. Other studies found that material uptake (endocytose) capacity of M cells is induced under various immunological conditions, e.g. a greater uptake of particles (0.1 micron, 1 micron and 10 micron diameter) has been demonstrated in the inflamed colonic mucosa of rats compared to non-ulcerated tissue [77,78] and inflamed oesophagus [79].

Diseases other than of gut origin also have marked effects on the ability of GIT to translocate particles. The absorption of 2-micron polystyrene particles from the PP of rats with experimentally induced diabetes is increased up to 100-fold (10% of the administered dose) compared to normal rats [80]. However, the diabetic rat displayed a 30% decrease in the systemic distribution of the particles. One possible explanation for this discrepancy is the increased density of the basal lamina underlying the GI mucosa of diabetic rats that may impede particle translocation into deeper villous regions. This uncoupling between enhanced intestinal absorption and reduced systemic dissemination has also been observed in dexamethasone treated rats [81].

From the literature cited above it is clear that engineered nanoparticles can be taken up via the intestinal tract. In general the intestinal uptake of particles is better understood and studied in more detail than pulmonary and skin uptake. Because of this advantage it is maybe possible to predict the behaviour of some particles in the intestines but precaution should be taken. For those nanoparticles designed to stabilise food or to deliver drug via intestinal uptake other, more demanding, rules exist and should be followed before marketing these compounds.

## 5. Skin

Skin is an important barrier, protecting against insult from the environment. The skin is structured in three layers: the epidermis, the dermis and the subcutaneous layer. The outer layer of the epidermis, the stratum corneum (SC), covers the entire outside of the body and only contains dead cells, which are strongly keratinized. For most chemicals the SC is the rate-limiting barrier to percutaneous absorption (penetration). The skin of most mammalian species is, on most parts of the body, covered with hair. At the sites, where hair follicles grow, the barrier capacity of the skin differs slightly from the "normal" stratified squamous epidermis. Most studies concerning penetration of materials into the skin have focussed on whether or not drugs penetrate through the skin using different formulations containing chemicals and/or particulate materials as a vehicle. The main types of particulate materials commonly used are: liposomes; solid poorly soluble materials such as TiO2 and polymer particulates and submicron emulsion particles such as solid lipid nanoparticles. The penetration of these particulate carriers has not been studied in detail.

TiO2 particles are often used in sunscreens to absorb UV light and therefore to protect skin against sunburn or genetic damage. It has been reported by Lademann *et al *in [82] that micrometer-sized particles of TiO2 get through the human stratum corneum and even into some hair follicles – including their deeper parts. However, the authors did not interpret this observation as penetration into living layers of the skin, since this part of the follicular channel (the acroinfundibulum) is covered with a horny layer barrier too [82]. A different interpretation has been suggested in a recent review by Kreilgaard [83], who argued that "very small titanium dioxide particles (e. g. 5–20 nm) penetrate into the skin and can interact with the immune system". Tinkle et al [84] demonstrated that 0.5- and 1.0 micron particles, in conjunction with motion, penetrate the stratum corneum of human skin and reach the epidermis and, occasionally, the dermis. The authors hypothesised that the lipid layers within the cells of the stratum corneum form a pathway by which the particles can move [85] into the skin and be phagocytized by the Langerhans cells. In this study the penetration of particles is limited to particle diameter of 1 micron or less. Nevertheless, other studies reported penetration through the skin using particles with diameters of 3–8 micron [86,87,82] but only limited penetration was found often clustered at the hair follicle (see above).

Penetration of non-metallic solid materials such as biodegradable poly(D,L-lactic-co-glycolic acid (PLGA) microparticles, 1 to 10 micron with a mean diameter of 4.61 ± 0.8 micron was studied after application on to porcine skin. The number of microparticles in the skin decreased with the depth (measured from the airside towards the subcutaneous layer). At 120 micron depth (where viable dermis present) a relatively high number of particles was found, at 400 micron (dermis) some micro-particles were still seen. At a depth of 500 micron no microparticles were found [88]. In the skin of individuals, who had an impaired lymphatic drainage of the lower legs, soil microparticles, frequently 0.4–0.5 micron but as larger particles of 25 micron diameter, were found in the in the dermis of the foot in a patient with endemic elephantiasis. The particles are seen to be in the phagosomes of macrophages or in the cytoplasm of other cells. The failure to conduct lymph to the node produces a permanent deposit of silica in the dermal tissues (a parallel is drawn with similar deposits in the lung in pneumoconiosis). This indicates that soil particles penetrate through (damaged) skin, most probably in every individual, and normally are removed via the lymphatic system [89,90]. Liposomes penetrate the skin in a size dependent manner. Micro-sized, and even submicron sized, liposomes do not easily penetrate into the viable epidermis, while liposomes with an average diameter of 272 nm can reach into the viable epidermis and some are found in the dermis. Smaller sized liposomes of 116 and 71 nm were found in higher concentration in the dermis.

Emzaloid™ particles, a type of submicron emulsion particle such as liposomes and nonionic surfactant vesicles (niosomes), with a diameter of 50 nm to 1 micron, were detected in the epidermis in association with the cell membranes after application to human skin [91]. The authors suggested that single molecules, which make up the particles, may penetrate the intercellular spaces and, at certain regions in the stratum corneum, are able to accumulate and reform into micro spheres. In a subsequent experiment, it was shown that the used formulation allowed penetration of the spheres into melanoma cells, even to the nucleus [92].

A recent review by Hostynek [93] stated that the uptake of metals through the skin is complex, because of both exogenous factors (e.g. dose, vehicle, protein reactivity, valence) and endogenous factors (e.g. age of skin, anatomical site, homeostatic control). Attempts to define rules governing skin penetration to give predictive quantitative structure-diffusion relationships for metallic elements for risk assessment purposes have been unsuccessful, and penetration of the skin still needs to be determined separately for each metal species, either by in vitro or in vivo assays.

Only limited literature on nanoparticles penetrating the skin is available, but some conclusions can already be drawn. Firstly, penetration of the skin barrier is size dependent, nano-sized particles are more likely to enter more deeply into the skin than larger ones. Secondly, different types of particles are found in the deeper layers of the skin and at present it is impossible to predict the behaviour of a particle in the skin. And finally, materials, which can dissolve or leach from a particle (e.g. metals), or break into smaller parts (e.g. Emzaloid™ particles), can possibly penetrate the skin. We did not find any direct indication that particles, that had penetrated the skin, also entered the systemic circulation. The observation that particles in the skin can be phagositized by macrophages, Langerhans cells or other cells is a possible road towards skin sensitisation. Tinkle *et al *[84] have shown that topical application of beryllium, to C3H mice, generated beryllium-specific sensitisation. These data are consistent with the development of a hapten-specific, cell-mediated immune response.

### 5.1 Mechanical irritation of skin

Glass fibres and Rockwool fibres are widely distributed man-made mineral fibres because of their multiple applications, mainly as insulation materials, which have become important for replacing asbestos fibres. In contact with the skin, these fibres can induce dermatitis through the mechanical irritation. Why these fibres are such strong irritant has not been examined in detail. In occlusion irritant patch tests in humans it was found that Rockwool fibres with a diameter of 4.20 ± 1.96 micron were more irritating than those with a mean diameter of 3.20 ± 1.50 micron. The fact that "small" fibres can cause strong skin irritation has been known for a long time, e.g. itching powder. It is also commonly accepted that some types of man made fibres can easily induce non-allergic dermatitis. Although this is common knowledge, it is not clear what makes these fibres irritants. In search for reports on skin irritation caused by fibres with a diameter of < 100 nm no information could be found, indicating that more research is needed.

## 6. Body distribution and systemic effects of particulates

The body distribution of particles is strongly dependent on their surface characteristics. For example, coating poly(methyl methacrylate) nanoparticles with different types and concentrations of surfactants significantly changes their body distribution [116]. Coating these nanoparticles with ≥ 0.1 % poloxamine 908 reduces their liver concentration significantly (from 75 to 13 % of total amount of particles administrated) 30 min after intravenous injection. Another surfactant, polysorbate 80, was effective above 0.5%. A different report [94] shows that modification of the nanoparticle surface with a cationic compound, didodecyldimethylammonium bromide (DMAB), facilitates the arterial uptake 7–10-fold. The authors noted that the DMAB surface modified nanoparticles had a zeta potential of +22.1 +/- 3.2 mV (mean +/- sem, n = 5) which is significant different from the original nanoparticles which had a zeta potential of -27.8 +/- 0.5 mV (mean +/- sem, n = 5). The mechanism for the altered biological behaviour is rather unclear, but surface modifications have potential applications for intra-arterial drug delivery.

Oral uptake (gavage) of polystyrene spheres of different sizes (50 nm to 3 micron) in female Sprague Dawley rats (for 10 days at a dose of 1.25 mg/kg/day) resulted in systemic distribution of the nanoparticles. About 7% (50 nm) and 4% (100 nm), was found in the liver, spleen, blood and bone marrow. Particles larger than 100 nm did not reach the bone marrow and those larger than 300 nm were absent from blood. No particles were detected in heart or lung tissue [75].

Irrespective of the uptake route, the body distribution of particles, is most dependent on the surface characteristics and the size of the particles. It is an important issue in drug-design in order to help to deliver medication to the right target. In unintentional uptake of nanoparticles these characteristics can strongly influence the accumulation of a specific type of particle in the particular body site.

### 6.1 Nanoparticles, thrombosis and lung inflammation

Epidemiological studies have reported a close association between particulate air pollution and cardiovascular adverse effects such as myocardial infarction [95]. The latter results from rupture of an atherosclerotic plaque in the coronary artery, followed by rapid thrombus growth caused by exposure of highly reactive subendothelial structures to circulating blood, thus leading to additional or complete obstruction of the blood vessel. Nemmar *et al *[96] studied the possible effects of particles on haemostasis, focusing on thrombus formation as a relevant endpoint. Polystyrene particles of 60 nm diameter (surface modifications: neutral, negative or positive charged) have a direct effect on haemostasis by the intravenous injection. Positively charged amine-particles led to a marked increase in prothrombotic tendency, resulting from platelet activation. A similar effect could be obtained after the intratracheal administration of these positively charged polystyrene particles, which also caused lung inflammation [97]. It is important to indicate that the pulmonary instillation of larger (400 nm) positive particles caused a definite pulmonary inflammation (of similar intensity to 60 nm particles), but they did not lead to a peripheral thrombosis within the first hour of exposure. This lack of effect of the larger particles on thrombosis, despite their marked effect on pulmonary inflammation, suggests that pulmonary inflammation by itself was insufficient to influence peripheral thrombosis. Consequently, the effect found with the smaller, ultrafine particles is most probably due, at least in part, to their systemic translocation from the lung into the blood.

Pollutant particles such as diesel exhaust particles (DEP), may cause a marked pulmonary inflammation within an hour after their deposition in the lungs. Moreover, intratracheal instillation of DEP promotes femoral venous and arterial thrombosis in a dose-dependent manner, already starting at a dose of 5 μg per hamster (appr. 50 μg/kg). Subsequent experiments showed that prothrombotic effects persisted at 6 h and 24 h after instillation (50 μg/animal) and confirmed that peripheral thrombosis and pulmonary inflammation are not always associated [97]. Solid inhaled particles are a risk for those who suffer from cardiovascular disease. Experimental data indicate that many inhaled particles can affect cardiovascular parameters, via pulmonary inflammation. Nano-sized particles, after passage in the circulation, can also play a direct role in e.g. thrombogenisis.

Epidemiologic studies have provided valuable information on the adverse health effects of particulate air pollution in the community, indicating that nanoparticles act as an important environmental risk factor for cardiopulmonary mortality. Particle-induced pulmonary and systemic inflammation, accelerated atherosclerosis, and altered cardiac autonomic function may be part of the patho-physiological pathways, linking particulate air pollution with cardiovascular mortality. Also, it has been shown that particles deposited in the alveoli lead to activation of cytokine production by alveolar macrophages and epithelial cells and to recruitment of inflammatory cells. An increase in plasma viscosity, fibrinogen and C-reactive protein has been observed in samples of randomly selected healthy adults in association with particulate air pollution [95,98,99].

### 6.2 Nanoparticles and cellular uptake

A number of reports on cellular uptake of micro- and nano- sized particles has been published. Reports on particle uptake by endothelial cells [100,101], pulmonary epithelium [102,79,103,59], intestinal epithelium [51,79] alveolar macrophages [104-107,57], other macrophages [89,108,76,109], nerve cells [110] and other cells[111] are available. This is an expected phenomenon for phagocytic cells (macrophages) and cells that function as a barrier and/or transport for (large) compounds. Except for macrophages, the health effects of cellular uptake of nanoparticles have not been studied in depth.

### 6.3 Nanoparticles and the blood-brain barrier

One of the promising alleys of nanotechnology is organ- or cell- specific drug delivery mediated by nanoparticles [112-114]. It is expected that transport of nanoparticles across the blood-brain barrier (BBB) is possible by either passive diffusion or by carrier-mediated endocytosis. Coating of particles with polysorbates (e.g. polysorbate-80) results in anchoring of apolipoprotein E (apo E) or other blood components. Surface modified particles seem to mimic LDL particles and can interact with the LDL receptor leading to uptake by endothelial cells. Hereafter, the drug (which was loaded in the particle) may be released in these cells and diffuse into the brain interior or the particles may be trans-cytosed.

Also, other processes such as tight junction modulation or P-glycoprotein (Pgp) inhibition also may occur [115]. Oberdörster et al 2002 reported the translocation of inhaled nanoparticles via the olfactory nerves [56]. Drug delivery systems crossing the BBB are certainly welcome, but this also implicates that unintended passage through the BBB is possible; therefore good safety evaluations are needed.

### 6.4. Nanoparticles and oxidative stress

It has been shown that nanoparticles, that enter the liver, can induce oxidative stress locally. A single (one day; 20 and 100 mg/kg) and repeated (14 days) intravenous administration of poly-isobutyl cyanoacrylate (PIBCA, a biodegradable particle) or polystyrene (PS, not biodegradable) nanoparticles induced a depletion of reduced glutathione (GSH) and oxidised glutathione (GSSG) levels in the liver, as well as inhibition of superoxide dismutase (SOD) activity and a slight increase in catalase activity. The nanoparticles did not distribute in the hepatocytes, implicating that the oxidative species most probably were produced by activated hepatic macrophages, after nanoparticle phagocytosis.

Uptake of polymeric nanoparticles by Kupffer cells in the liver induces modifications in hepatocyte antioxidant systems, probably due to the production of radical oxygen species [108]. We have discussed above that nano-sized particles in the lung can induce, via the pulmonary inflammatory response as well as via spontaneously surface related reactions, oxidative stress. Besides pulmonary studies, not many have studied particle-induced oxidative stress in tissues. However, the authors [108] reported that the depletion in glutathione was not sufficient enough to initiate significant hepatocytic damage (no lipid peroxidation). It needs to be stressed that long-term studies are needed to prove the safe use of these nanoparticles because chronic depletion of the anti-oxidant defence can lead to severe health problems.

## 7. Differences in conditions between the lung and intestinal tract

Although the contact with nanomaterials in the lungs and intestinal tract shows many similarities important differences between inhalation and ingestion of nanomaterials exist from the toxicological point of view. In the intestinal tract a complex mix of compounds – such as secreted enzymes, ingested food, bacteria of the gut flora, etc – is present, which can interact with the ingested nanomaterial. Non-specific interaction often reduces the toxicity of the ingested material. It has been described that *in vitro *particles are less cytotoxic when dosed in a medium with high protein content. In the lungs, mucus or surfactant is present, in which antioxidants are present, but these can be easily neutralised when a high number of oxidative compounds is inhaled.

The transit through the intestinal tract is a relatively fast process, the continuous decay and renewal of the epithelium makes sure that nanomaterials will not remain long in the intestinal tract. The presence of solid material in the lumen of the intestines will not automatically induce an inflammatory response. Inhaled materials < 10 micron and > 5 micron will not enter the alveolar spaces of the lungs, and therefore these will be cleared easily in healthy persons via the muco-ciliary escalator. Particles that are smaller than 5 micron will deposit in the alveolar space via Brownian movement. In the alveoli, water insoluble materials can only be removed via phagocytosis by macrophages or other cells, or via transportation through the epithelium to the interstitium or systemic circulation. These processes are often accompanied by the onset of (persistent) inflammation. The particles themselves can – depending on the physical-chemical characteristics of the material – remain for a long period in the alveoli.

In the intestinal tract, the ingested materials are stressed from acidic (stomach) to basic conditions. The shift in pH markedly changes the solubility and the ionic state of the material via changing the surface characteristics. In the lungs, the milieu of the lumen is more constant.

## 8. Conclusions

Particles in the nano-size range can certainly enter the human body via the lungs and the intestines; penetration via the skin is less evident. It is possible that some particles can penetrate deep into the dermis. The chances of penetration depend on the size and surface properties of the particles and also on the point of contact in the lung, intestines or skin. After the penetration, the distribution of the particles in the body is a strong function of the surface characteristics of the particles. A critical size might exist beyond which the movement of the nanoparticles in parts of the body is restricted. The pharmaco-kinetic behaviour of different types of nanoparticles requires detailed investigation and a database of health risks associated with different nanoparticles (e.g. target organs, tissue or cells) should be created. The presence of the contaminates, such as metal catalysts present in nanotubes, and their role in the observed health effects should be considered along with the health effect of the nanomaterials.

The increased risk of cardiopulmonary diseases requires specific measures to be taken for every newly produced nanoparticle. There is no universal "nanoparticle" to fit all the cases, each nanomaterial should be treated individually when health risks are expected. The tests currently used to test the safety of materials should be applicable to identify hazardous nanoparticles. Proven otherwise, it would be a challenge for industry, legislators and risk assessors to construct a set of high throughput and low cost tests for nanoparticles without reducing the efficiency and reliability of the risk assessment. Nanoparticles designed for drug delivery or as food components need special attention.
